# Domestic violence as a risk factor of maternal filicide

**DOI:** 10.1007/s00737-024-01430-8

**Published:** 2024-02-15

**Authors:** Julia Vileisis, Brooke Laufer

**Affiliations:** 1 Department of Psychiatry and Behavioral Sciences, Albert Einstein College of Medicine, Bronx, NY USA; 2 Private Practice, Independent Scholar, Evanston, IL USA; 3 Department of Psychiatry and Behavioral Sciences, Montefiore Medical Center, Bronx, NY, USA

**Keywords:** Filicide, Infanticide, Intimate partner violence, Domestic violence, Postpartum depression, Battered woman syndrome

## Abstract

**Purpose:**

This paper will investigate precursors to maternal filicide, focusing on domestic violence. While psychosis is often well described, less frequently explored are the connections between prior trauma, domestic violence, depression, and filicide. We will discuss reasons why a woman may not disclose domestic violence and suggest possible areas for intervention.

**Methods:**

We present a case involving domestic violence, its impact on mental health, and eventual filicide. We then present an alternative scenario of the same case where filicide is considered, but is avoided.

**Results:**

The case of the mother who experienced domestic violence and was accused and sentenced for filicide is seen in greater relief by presenting the case in an alternative scenario with effective interventions. It is clear the availability and the ability to access community supports, such as obstetric and pediatric screening, psychiatric treatment, domestic violence shelters, intimate partner violence outreach services, parenting support groups, and hospital social work case management, tragedies such as filicide can be prevented.

**Conclusion:**

Traumatic early childhood experiences predispose people to a stress–response system that is more prone to inactivity and impulsivity. This can cause women in domestic violence relationships to stay, limit their options for family planning, become increasingly depressed, not seek community support, and risk impulsive action of harming their child. This risk can be mitigated by building stable relationships with their medical team, treating depression, connecting with home visitation programs, and being empowered to access contraception.

## Introduction

Domestic violence (DV) is a highly correlated risk factor of maternal filicide and should potentially be viewed as a precursor (Friedman et al. 2005). Infanticide is the murder of an infant under 1 year old, whereas filicide refers to the death of a child between the ages of 1 year old and 18 years old (Hatters Friedman & Resnick 2007). Neonaticide is the murder of a child in the first 24 h of life (Friedman et al. 2005). Both authors are clinicians who have worked with mothers experiencing domestic violence and who either have all the risk factors for or who have committed infanticide and filicide. This article will present one of those cases in order to examine the ways DV in a mother’s life affects her mental health and the devastating event of a child’s death caused by their mother. Our case considers a mother who experienced severe DV and was accused and sentenced for filicide.

## Literature review

### Mother’s characteristics

There are overlapping characteristics of mothers who commit neonaticide, infanticide, and filicide. While these events occur across race and socioeconomic status, the typical profile of an infanticidal mother includes women who have disadvantaged socioeconomic backgrounds, are unemployed, and have a mean age in the early 20s (Baek et al. 2019; Friedman et al. 2005; Hatters Friedman & Resnick 2007). Filicidal mothers tend to have disadvantaged socioeconomic backgrounds, are socially isolated, are full-time caregivers, have conflicts with family members, and are victims of domestic violence (Bourget et al. 2007; Friedman et al. 2005; Hatters Friedman & Resnick 2007; Mailloux. 2014; Oberman & Meyer 2008). Most studies find that about half of the mothers who have committed infanticide have experienced psychosis (Raymond et al. 2021; Porter and Gavin 2010). Neonaticidal mothers are often young, unmarried women who receive no prenatal care (Bourget et al. 2007; Friedman et al. 2005). Postpartum psychosis, race, class, and status, though highly relevant to the occurrence of maternal filicide, will not be the focus of this paper in order for the authors to highlight DV.

### The role of intimate partner violence

Domestic violence is a pattern of abusive behavior in which a person uses coercion, deception, harassment, humiliation, manipulation, or force to establish power and control over an intimate partner. Economic, emotional, psychological, physical, sexual, and verbal tactics are used by perpetrators to sustain that control and achieve submission in their partners. In the USA, the Centers for Disease Control and Prevention has redefined the term “domestic violence” and uses the term “intimate partner violence” (IPV), which acknowledges violence between same-sex partners and male victims and that violence can occur in a wide range of living arrangements. In this paper, the term domestic violence will be used interchangeably with the term intimate partner violence. Socioeconomic disadvantages increase the risk of being the victim of violent crimes. IPV occurs at different rates by race and ethnicity in the USA with 63.8% of non-Hispanic multiracial women, 57.7% of non-Hispanic American Indian or Alaska Native women, 53.6% of non-Hispanic Black women, 48.4% non-Hispanic white women, 42.1% of Hispanic women, and 27.2% of non-Hispanic Asian or Pacific Islander women reported this lifetime occurrence (Leemis et al. 2022).

Domestic violence impacts the brain and behavior. Battered woman syndrome (BWS) is a psychological condition that describes a pattern of behavior that develops in victims of DV as a result of serious, long-term abuse. BWS is dangerous primarily because it can lead to “learned helplessness” – or psychological paralysis – where the victim becomes so depressed, defeated, and passive that she believes she is incapable of leaving her abuser. Symptoms include refusing to leave the relationship, believing that the other person is powerful or knows everything, idealizing the person who carried out the abuse, and believing that they deserve the abuse.

### Domestic violence and infanticide

Studies that find the least correlation between DV and infanticide concur that about 50% of infanticidal mothers were living in violent relationships (Meyer et al. 2001; Sidebotham & Retzer 2018). In yet another study of infanticidal mothers, researchers found that all of the women in the sample were victims of violence by their partners (Kachaeva 2015). The women who had committed infanticide displayed “pathological altruistic motivation,” meaning they killed their children for reasons of saving them as they knew the world to be a violent one (Kachaeva 2015).

### Domestic violence and filicide

When investigating filicide, domestic violence toward the mother was reported in up to 79% of cases of child death (Quigley 2007). One study found that 46% of mothers who committed filicide in Italy were in an IPV relationship (Giacco et al. 2023).

### Domestic violence and child abuse

There are overlapping incidents of domestic violence and child abuse. There is a correlation between women who commit filicide and have their own childhood history of experiencing abuse (Bourget et al. 2007). In Northern Italy, 29% of mothers who committed filicide experienced abuse during their own upbringing, whereas in France, this occurred in over 70% of cases (Giacco et al. 2023; Raymond et al., 202). Later in life, women who experienced childhood abuse are often found to be in romantic relationships where their partner abuses them and their children (Kelleher et al. 2006).

### Failure to protect

Often, abused mothers are accused of failing to protect their children from harm at the hands of their partners. Much of what is known about battered women would suggest that these women are caught in the cycle of an abusive relationship and are unable to act to protect themselves or their children. A failure to leave is conflated with a failure to protect, but instead of reflecting mental illness or apathy, these cases generally arise out of a battered woman’s rational fear of being required to manage alone with limited economic and social support or the risk of being killed for attempting to leave the relationship.

### Domestic violence and trauma

Often, domestic violence is not prevented nor treated because women do not disclose their experiences to healthcare providers or police officers. Domestic violence is traumatic, and as renowned trauma expert Dr. Bessel Van Der Kolk (2014) states, “Traumatized people often have enormous difficulty telling other people what has happened. Their bodies experience terror, rage, and helplessness, as well as an impulse to fight or flee, but these feelings are impossible to articulate.” Additionally, a battered woman may not share her story due to shame or cultural beliefs that beholden her to keep her problems within the home. Finally, although the WHO reports it is an expectation of Member States to protect the lives of infants, a battered mother may perceive disclosure as futile, as neither law enforcement nor the legal world has regularly responded to DV (Cheng et al. 2022; WHO, 2019). In fact, in circumstances where IPV is a mitigating factor when a woman is accused of a crime herself, it is likely the information will be used against a mother, as she is perceived as having failed to protect herself and her children, furthering herself as an irresponsible mother.

### Domestic violence and depression

There is a significant correlation between IPV and postpartum depression (PPD); both contribute to incidents of child abuse and filicide (Hatters Friedman & Resnick 2007). Regardless of pregnancy or parenting status, women who experience IPV are more likely to develop symptoms of depression, anxiety, and other mental health conditions than women who have not been abused (Stewart and Vigod 2017; Rodríguez et al. 2010). Moreover, PPD increases the chances of harm coming to an infant (Raymond et al. 2021); chronic mental illness is a substantial risk factor for infanticide (Spinelli 2003).

### Domestic violence and pregnancy

Many women report that IPV started or intensified when they became pregnant (Goodman 2021). Despite the increasing risk of violence as pregnancy progresses, many obstetrical practices only screen at the first visit, if at all (Alhusen et al. 2015). For women who experience IPV during pregnancy, the odds of developing PPD are increased 2.73 times those who do not experience IPV (Wei et al. 2023). Similarly, studies among Latinas indicated IPV increased the risk of depression in the perinatal time (Rodríguez et al. 2010). Additionally, the impact of IPV on PPD was higher in low- and middle-income regions compared to high-income regions (Wei et al. 2023). Notably, the population with the highest rates of comorbid PPD and IPV in the USA are Black teenage moms (Kornfeld et al. 2012). Additionally, women who are abused during pregnancy are more likely to receive no prenatal care or to delay care, setting both mother and child up for poor health outcomes (Alhusen et al. 2015).

## Case analysis

Ruth (name changed) grew up in a violent and impoverished housing complex with a severely alcoholic mother and a father who regularly “whooped” (often with a wire) her and her 4 siblings. She became pregnant at 13 years old, then miscarried while 6 months pregnant from her sister kicking her in the stomach, after which Ruth attempted suicide with pills, which she repeated on and off throughout her life. Ruth reported that she was never treated or hospitalized, nor did she remember ever having sex health education or interaction with a school social worker.

Ruth became pregnant again at 18 years old and was kicked out of her father’s house. Ruth moved in with a boyfriend who was extremely physically abusive. After having her child, she took in a toddler—a common occurrence in her community—who had prenatal drug and alcohol exposure and developmental delays and whose mother was incarcerated. Ruth was particularly dedicated to this child, who was partially blind, helping her learn to walk and hold a bottle. Ruth eventually adopted the child. At 28, she became pregnant for the third time and was anxious about miscarriage due to the domestic violence she was experiencing. Ruth reported that she had never heard of local domestic violence shelters.

While Ruth was pregnant, the boyfriend’s coercive behavior increased toward her, becoming extreme. He was jealous and controlling and, at his whim, physically and sexually assaulted Ruth. He kept Ruth from friends and family, rarely allowing her to leave their apartment without “permission.” Several times, the boyfriend assaulted her in a way that convinced her she was miscarrying the child. He also physically abused the young children, and Ruth often attempted to protect them. He insisted the children be quiet and behaved well, but was never a caretaker for them himself. Ruth believed he was abusing and selling drugs. Ruth’s depression and suicidality increased.

Caring for her biological child and disabled adopted child, at 8 months pregnant, Ruth was exhausted, depressed, and literally beaten down by her boyfriend. One afternoon, the adopted daughter put her hand on a hot burner. Ruth engaged in corporal punishment and lost control. At the end of the whooping, the child was unconscious, and Ruth rushed her to a hospital that served mostly white patients. Ruth transparently reported the events. The child was pronounced dead, and Ruth was arrested. In what suggests both sexist and racial profiling, Ruth, a Black mother, was presumed sole guardian, and the White male police never questioned the abusive partner for his possible role. Ruth was sentenced to prison shortly after she delivered her baby. Her oldest child and newborn went to be cared for by her sister, while Ruth was sentenced to 55 years in prison.

No one in Ruth’s life addressed or acknowledged the abuse she received, and neither had Ruth, as it was an accepted part of her family culture. The effects of that abuse were stored in her body, sticks of dynamite with which she entered adulthood, when each additional episode of intimate partner abuse added a stick to her pile until there was a trigger at a vulnerable moment, and Ruth snapped. At the time she was 8 months pregnant, exhausted and hopeless, several years entrapped by an abusive partner, and ready to end her life.

Ruth overreacted to the threat of a 4-year-old girl innocently, but dangerously, playing with a stove. Ruth’s traumatic memories and nervous system caused her to enter fight mode and respond disproportionately. She experienced rage and an inflated sense of strength and had very few resources to manage the moment when her dynamite was lit, and she lost control. The state that overcame Ruth is indicative of a flashback when one is dissociated from present and current events, possessed by the nervous system’s dysregulated rage and fight reaction to cumulative, past trauma and overwhelming fear.

## Alternative case of Ruth

An alternative scenario illustrates areas of possible interventions for Ruth, ultimately preventing the death of her child. When Ruth was a child, there were signs of physical abuse and neglect about which teachers became concerned. Although the school filed a case with Child Protective Services, there appeared to be no significant change in Ruth’s living situation. However, Ruth developed a positive view of the caseworker who she thought was friendly. Also, Ruth’s second-grade teacher made additional efforts to speak with Ruth individually. She found that Ruth was particularly skilled in drawing. Often, the teacher’s enthusiasm about her artwork was the only positive feedback Ruth received from an adult. When Ruth became pregnant at the age of 13, she stopped attending school. At the age of 18, she became pregnant again and moved in with a violent boyfriend. She began thinking of her motherly second-grade teacher and went to visit her. Ruth did not reveal the violence she experienced, but her teacher encouraged her to attend prenatal visits. At these visits, Ruth learned of contraception and, after delivery, received an intrauterine device. Because of an old medical record concerning abuse, a history of a late-term miscarriage at a very young age, and that she was late to prenatal care, her obstetrician recommended she speak with their social worker. To address her depression, Ruth agreed to regular visits with the social worker as she had positive memories of her case worker. Again, she did not reveal she was currently experiencing DV, but described childhood abuse from her father. The astute social worker knew of the correlation between experiencing childhood abuse and DV and occasionally mentioned resources for those experiencing DV. The abuse continued to escalate, and several months after delivery, Ruth realized her child would experience a childhood even worse than Ruth had. Although she had thoughts of filicide, she did not see this as the only option, and instead, Ruth got the courage to call the DV hotline her social worker had discussed, and she entered a DV shelter. She continued contact with her former teacher who encouraged her to enroll in GED classes, which allowed her to support herself and her child.

## Interventions and advocacy for perinatal mental health services

It is clear from the above alternative case scenario that interventions along the trajectory of a woman’s life can affect its outcome. Community support services such as Child Protective Services, DV shelters and hotlines, counseling, and case management can protect the life of a child. Communication between healthcare providers and community support requires improvement. Healthcare workers, along with the police force, require regular education on signs and symptoms of IPV and depression along with knowledge of local resources, with whom they have established relationships, and treatments for depression. Counseling services are the recommended form of prevention and treatment for perinatal depression and should address IPV concerns (Curry et al. 2019). Moreover, alliances with international organizations such as Postpartum Support International and the International Marcé Society for Perinatal Mental Health can offer training and education to professionals and laypersons on these important subjects.

## Limitations

Research is limited as filicide is a relatively rare occurrence, and many instances of infant and child deaths go unreported (Craig 2004). Additionally, many studies investigate incarcerated or psychiatric populations who have committed filicide, yet 16–29% of mothers who have committed filicide died by suicide and are therefore not represented (Friedman et al. 2005). There are several limitations to our discussion; namely, it does not qualify for statistical tests as it requires a larger sample size to ensure that the sample is considered representative of a population and that the statistical result can be generalized to a larger population. As a qualitative study, it was limited to one case and relied on theoretical data (an alternative scenario) to highlight interventions. Future research on the correlation between DV and neonaticide, infanticide, and filicide is required, along with intervention studies.

## Conclusion

Mothers who find themselves in the unthinkable circumstances of killing their own child are most often in the throes of PPD and IPV. They have not planned nor preconceived their crime; most often, they have no criminal history, nor have they ever displayed signs of being a neglectful mother. A mother with very little support, under the control of a violent man, unable to care properly for her child, and afraid for her own life along with her child’s, with symptoms of fatigue, low self-worth, and suicidality, is in a perfect storm for an ultimate tragedy. Therefore, it is imperative that PPD and IPV are conditions that providers are able to identify, report, and treat. If law enforcement or psychiatric providers can at least recognize these comorbid warning signs, it is possible the horrific occurrences of filicide can be prevented.

The correlation between domestic violence and maternal filicide is complicated and may not be the same in every situation. One understanding of this pathway, from domestic violence to the murder of a child, is explained by complex trauma. A heightened level of complex trauma, such as physical or emotional abuse, neglect, caregiver substance abuse or mental illness, exposure to violence, and the accumulated burdens of family economic hardship, can lead to a toxic stress response. This stress–response system can disrupt the development of brain architecture and increase the risk for stress-related disease and cognitive impairment well into the adult years. Physician Gabor Maté (2018) has compiled a wealth of research about how isolation, trauma, and neglect harm the developing brain, including various parts of the prefrontal cortex that help us regulate our impulses and weigh the future outcomes of our actions. Dr. Maté explains that, in essence, the result later in life can be adults lacking not free will, but a “free won’t.” A history of trauma can mean we have particular difficulty interrupting impulses, such as the impulse to snap. Although a person’s snap into violence may come as a total surprise, in most cases, there is a psychological buildup to that point (Ash 2019).

Identifying signs of IPV, PPD, and suicidality and implementing interventions are paramount for authorities and providers in a mother’s life. Obstetric and other medical visits provide many opportunities for a woman experiencing IPV and depression to build relationships with her providers that can help her feel safe enough to disclose abuse. Visits may be one of the few reasons she may be allowed to leave the home. All providers that come into contact with a woman should collaborate regularly including obstetrics, midwifery, nursing, psychiatry, social work, and pediatrics. IPV should be universally screened for at every visit, both as the strength of the provider-patient relationship grows, but also as the risk of violence increases. Women should be referred to home visitation programs, as these have been shown to decrease IPV exposures (Alhusen et al. 2015). Because of the well-established bidirectional relationship of IPV and PPD, women should also be regularly screened for PPD and assessed for suicidality. Providers should be trained and feel confident in intervening, treating depression, and helping the woman to maximize access to community resources. Even prior to conception, women should be given education and access to contraception, which has been shown to reduce filicide (Friedman & Resnick 2009). Decreasing the devastating outcomes of IPV, including maternal filicide, is possible with the diligent efforts of those supporting women.
